# PCGF6/MAX/KDM5D facilitates MAZ/CDK4 axis expression and pRCC progression by hypomethylation of the DNA promoter

**DOI:** 10.1186/s13072-023-00483-w

**Published:** 2023-03-09

**Authors:** Meng Zhu, Ruo-Nan Zhang, Hong Zhang, Chang-bao Qu, Xiao-chong Zhang, Li-Xin Ren, Zhan Yang, Jun-Fei Gu

**Affiliations:** 1 Department of Urology, The Second Hospital of Hebei Medical University, 215 Heping West Road, Shijiazhuang, 050000 China; 2 School of Chinese Integrative Medicine, Hebei Medical University, Shijiazhuang, Hebei China; 3 Molecular Biology Laboratory, Talent and Academic Exchange Center, The Second Hospital of Hebei Medical University, Shijiazhang, China; 4 Clinical Laboratory, Xingtai People’s Hospital, Xingtai, China

**Keywords:** PCGF6, Myc-related zinc finger protein, Hypomethylation, Papillary renal cell carcinoma

## Abstract

Polycomb group RING finger protein 6 (PCGF6) plays an important role as a regulator of transcription in a variety of cellular processes, including tumorigenesis. However, the function and expression of PCGF6 in papillary RCC (pRCC) remain unclear. In the present study, we found that PCGF6 expression was significantly elevated in pRCC tissues, and high expression of PCGF6 was associated with poor survival of patients with pRCC. The overexpression of PCGF6 promoted while depletion of PCGF6 depressed the proliferation of pRCC cells in vitro. Interestingly, myc-related zinc finger protein (MAZ), a downstream molecular of PCGF6, was upregulated in pRCC with hypomethylation promoter. Mechanically, PCGF6 promoted MAZ expression by interacting with MAX and KDM5D to form a complex, and MAX recruited PCGF6 and KDM5D to the CpG island of the MAZ promoter and facilitated H3K4 histone demethylation. Furthermore, CDK4 was a downstream molecule of MAZ that participated in PCGF6/MAZ-regulated progression of pRCC. These results indicated that the upregulation of PCGF6 facilitated MAZ/CDK4 axis expression and pRCC progression by hypomethylation of the MAZ promoter. The PCGF6/MAZ/CDK4 regulatory axis may be a potential target for the treatment of ccRCC.

**Supplementary Information:**

The online version contains supplementary material available at 10.1186/s13072-023-00483-w.

## Introduction

Renal cell carcinoma (RCC) originates from the urothelium of the renal parenchyma, with a high incidence, accounting for about four-fifths of renal malignant tumors [1]. RCC ranks 10th in cancer incidence of all malignant tumors and is the most common malignant tumor of the urinary system [2]. Kidney cancer is divided into multiple tissue types, each with different characteristics that depend on genetic drivers [3]. The incidence of papillary RCC (pRCC) is the second most common after clear cell RCC (ccRCC), accounting for approximately 15% of RCCs [4]. pRCC is a heterogeneous tumor including two subtypes: pRCC1 and pRCC2 [5]. The prognosis of pRCC is significantly better than that of ccRCC without metastases in patients, and its 5-year tumor-specific survival rate is also higher than that of ccRCC [6]. Currently, there was no effective treatment for patients with advanced pRCC [7]. Therefore, the molecular mechanism of pRCC should be explored to help determine candidate biomarkers and therapeutic targets and develop new therapeutic methods.

Polycomb group RING finger protein 6 (PCGF6), also called as MBLR, RNF134, is a component of the Polycomb group family. PCGF6 was first identified in a polyprotein complex associated with the E2F6 transcription factor [8]. Some studies have found that PCGF6 acted as a transcription inhibitor by interacting with certain Polycomb group (PCG) proteins [9]. For example, the PCGF6-PRC1 complex remodeled through chromatin and mediated the monoubiquity of histone H2A “Lys-119”. Histone modifications inhibited gene expression [9]. In contrast, a recent study identified that PCGF6 as a transcriptional activator regulated stem cell pluripotency through super-enhancer-dependent chromatin interactions [10]. Interestingly, other studies have found that PCGF6 regulated histone H3K4Me3 levels and downstream gene expression by activating KDM5D histone demethylase [11]. PCGF6 and BMI1 promoted the development of colitis-related cancer in mice by regulating the expression of Reg3b which promoted proliferation and reduced apoptosis [12]. However, the role of PCGF6 in pRCC remains unclear.

Myc-related zinc finger protein (MAZ) was first identified as a transcription factor of MYC [13]. MAZ is commonly expressed in various tissues, usually in combination with GC-rich sequences, such as GGGAGGG or CCCTCCC, and plays a dual role of transcription initiation and termination in gene transcription [13, 14]. MAZ not only activated the gene expression of c-Myc, insulin, VEGF, PDGF, and RAS gene families [13, 15–18], but also terminated the transcription of eNOS and p53 [19, 20]. MAZ played a key role in the progression of prostate, colorectal, pancreatic, and breast cancers, among other cancers [15, 21–23]. MAZ transcription factor was the downstream target of the oncoprotein Cyr61/CCN1, which promoted the invasion of pancreatic cancer cells through the CRAF-ERK signal [22]. The two functions of FOXF2-mediated MAZ in basic breast cancer promoted proliferation and inhibited progression [23]. MAZ drove the tumor-specific expression of PPARγ1 in breast cancer cells [21]. MAZ was reported to be upregulated in ccRCC, and upregulated MAZ promotes cancer [24]. However, the expressed regulation and function of MAZ in pRCC are unknown.

This study showed the elevated levels of PCGF6 and MAZ in pRCC sample, and the upregulation of these genes were associated with poor patient survival. The upregulation of PCGF6 promoted the demethylation of H3K4me3 in CpG island of MAZ promoter. PCGF6 interacted with MAX and KDM5D to form a complex, and MAX recruited PCGF6 and KDM5D to the CpG island of the MAZ promoter and promoted histone demethylation. CDK4 was a downstream gene of MAZ and was involved in MAZ-induced pRCC progression. The PCGF6/MAZ/CDK4 signaling pathway demonstrated an important role in regulating its proliferation during pRCC, which provided a new potential therapeutic target for the treatment of pRCC.

## Materials and methods

### Tumor samples

The pRCC tissues and corresponding normal kidney tissues were brought from the Second Hospital of Hebei Medical University between June 2013 and February 2022. Patients underwent radical nephrectomy during hospitalization, and pathological analysis was performed. Written informed consent was obtained from all the patients enrolled in this study. The research protocol was approved by the Ethics Committee of the Second Hospital of Hebei Medical University (No. 2020-R373).

### Cell line, transfection, and vector construction

Human pRCC cell lines (i.e., SKRC-39, ACHN, Caki-2, and UOK-112) were purchased from ATCC (Maryland) for culture and were stored in the laboratory. The cells were cultured in low-glucose medium. Then, 8% FBS (Clark Bio) was added with 1% penicillin/streptomycin (Solarbio) to the low-glucose medium. The cells were cultured in a dressing box containing 5% CO_2_ and 95% air. Cells were transfected with Lipofectamine 2000 (Invitrogen, Thermo Fisher Scientific, Massachusetts, MA) according to the manufacturer’s operating manual. The lentiviral vectors were constructed for the experiments in the laboratory, including pLKO-shMAZ and pWPI-MAZ, and stored at − 80 °C. In addition, shPCGF6 and oePCGF6 lentiviral vectors were purchased from Hanbio Biotechnology Co, Ltd. (Shanghai, China).

### Total RNA extraction and RT-qPCR

RNAeasy Mini Elute Kit (QIAGEN, Germany) was used to isolate total RNA according to the commercial operation manual. Then, concentration and quality of RNA were detected using NanoDrop 2000 system. The first strand of cDNA was synthesized using M-MLV’s First Strand Kit (USA) according to the operation manual. The synthesized cDNA was diluted 5 to 10 times according to the amplification needs. The expression of mRNA was detected by RT-qPCR (Bio-Rad USA) in a CFX96 real-time system using the Monad kit (MonScript, Monad, China) with indicated primers (Additional file 1: Table S1). The expression of mRNA was normalized using *GAPDH* as an internal reference gene. The relative expression of mRNA was analyzed and calculated using the 2^−ΔΔCt^ formula [24].

### Western blot analysis

Proteins were detected in cells and tissues by Western blotting as described earlier [25]. Protein lysate was used to extract total protein from cells and frozen tissue samples. The protein concentration in the resulting protein samples was determined using a modified Lowry method. The proteins were then separated by SDS-PAGE and then transferred to PVDF membranes (Merck Millipore) using the semi-dry method. After blocked with 5% milk, the membrane was incubated with the primary antibody for 2–4 h at 37 °C. The primary antibodies were as follows: MAZ (1:1000, 21068-1-AP), PCGF6 (1:1000, 25814-1-AP), MAX (1:1000, ab199489), KDM5D (1:500, ab194288), CDK4 (1:1000, 11026-1-AP), H3K4me3 (1:1000, ab8580), and β-actin (1:5000, sc-47778). Then, the membrane was incubated with horseradish peroxidase (HRP)-conjugated secondary antibodies (1:10000, Rockland). The membrane was incubated with ECL Luminescent Fluid (WBKLS0500, Millipore) after washing. Then used eFusionCapt Advance Fx5 (Collégien, France) to capture and analyze all images.

### DNA methylation detection

Bisulfite sequencing was applied to detect the MAZ promoter region in pRCC tissue. Total DNA was isolated from tumor tissues and the EZ DNA Methylation-Lightning Kit (#D5030) was used to detect genomic DNA. The DNA was amplified by polymerase chain reaction (PCR) reaction, and the production was purified using the QIAquick PCR Extraction Kit (Germany). The purified BSP product was ligated with peasy-T5 vector and introduced into competent cells. The DNA plasmid from a single colony was extracted and sequenced directly.

### Immunohistochemistry

pRCC tissues with a slice thickness of 4 μm were roasted at 65 °C for 2 h, dewaxed and dehydrated, repaired under high pressure for 3 min, and then returned to room temperature. The instructions of the immunohistochemistry (SP0041, Solarbio) kit were followed until the tissue was incubated with the antibody MAZ (1:100, 21068-1-AP). Seven fields of view were randomly selected, photographed with a microscope. The percentage of brown particles in each field of view was quantified using Integrated Performance Primitives (version 6.0; Intel Corporation, CA).

### Morphometry and histology

Healthy kidney tissues and fresh pRCC tissues were washed with 0.9% saline surface blood. Tissues were fixed in formalin fixative for 48 h. Conventional paraffin-embedded tissue was used for the above. The embedded tissue was histologically sectioned to a thickness of 5 μm and performed the HE staining. The images of HE staining were acquired using a Leica microscope (DM6000B; Leica) and quantified using the Leica Application Suite (version 4.4; Leica).

### Cell viability assay

MTT colorimetric assay was performed to measure cell viability. First, 5000 cells of SW839 and Caki-2 were planted into 96-well plates. Transfections were performed in groups of experiments or stimulated with AZD6244 for 24 h. Cell medium was discarded and replaced with MTT reagent (Sigma-Aldrich) and serum-free medium in 96-well plates. Then, the 96-well plate was incubated for 3 to 4 h. A microplate reader (Massachusetts, USA) was used to measure the absorbance of each space.

### Colony formation assay

Hundred cells were seeded for each well of a six-well plate. The cells were cultured with 8% medium in an incubator at 37 °C for 1 week and then fixed with methanol solution. The six-well plate colonies were then stained with 0.25% crystal violet solution. Finally, the number of colonies were counted and analyzed under the microscope.

### Chromatin immunoprecipitation assay

The steps were followed as described for the previous ChIP assay [26]. The cultured cells were first fixed with formaldehyde. Then, cells were sonicated and fragments of 400–600 bp in size were obtained to cross-link chromatin. Samples were diluted and pretreated with blocked A-Sepharose beads for 30 min. Supernatants were immunoprecipitated overnight with anti-H3K36me2 or anti-IgG antibodies. Finally, the beads were uncross-linked from the protein and the occupancy of H3K36me2 on the MAZ CpG promoter was detected by RT-qPCR.

### Co-immunoprecipitation assay

Magnetic beads (MCE) were incubated with the antibody to form a magnetic bead–antibody complex. The collected cells were lysed on ice, and an equal amount of protein solution was used for co-immunoprecipitation (CoIP) and then incubated with rotation to form magnetic bead–antigen–antibody complexes. The supernatant was discarded after washing four times with buffer. An eluate of 35 ul was added to the magnetic bead–antigen–antibody complex, and cooked at 100 °C for 10 min. Finally, protein interactions were analyzed by Western blot or mass spectrometry [27].

### Xenograft animal model

In vivo tumor growth assays were performed [25, 27, 28]. Male BALB/c nude mice (4–6 weeks) in this study were purchased from Vital River Laboratory Animal Technology Co., Ltd. (Beijing). Caki-2 cells were collected stably infected with LV-shMAZ or LV-shPCGF6. All cells were harvested by trypsinization; 5 × 106 cells were mixed with 50% Matrigel (BD, NJ) and 50% serum-free medium and injected subcutaneously into the right posterior flank of the mice. The length and width of subcutaneous xenograft tumors in mice were measured twice a week. The mouse tumor volume was calculated by (length × width^2^)/2.

### Statistical analysis

We used Student’s *t*-test which was performed to analyze the differences between the two groups of data. All data were expressed as mean ± standard error. Spearman’s correlation analysis was performed to evaluate correlations. *p*-values of less than 0.05 were used to indicate statistical significance. Prism GraphPad Software was used for graphing and statistical analysis.

## Results

### PCGF6 expression increases in pRCC and correlated with poor prognosis

**Fig. 1:**
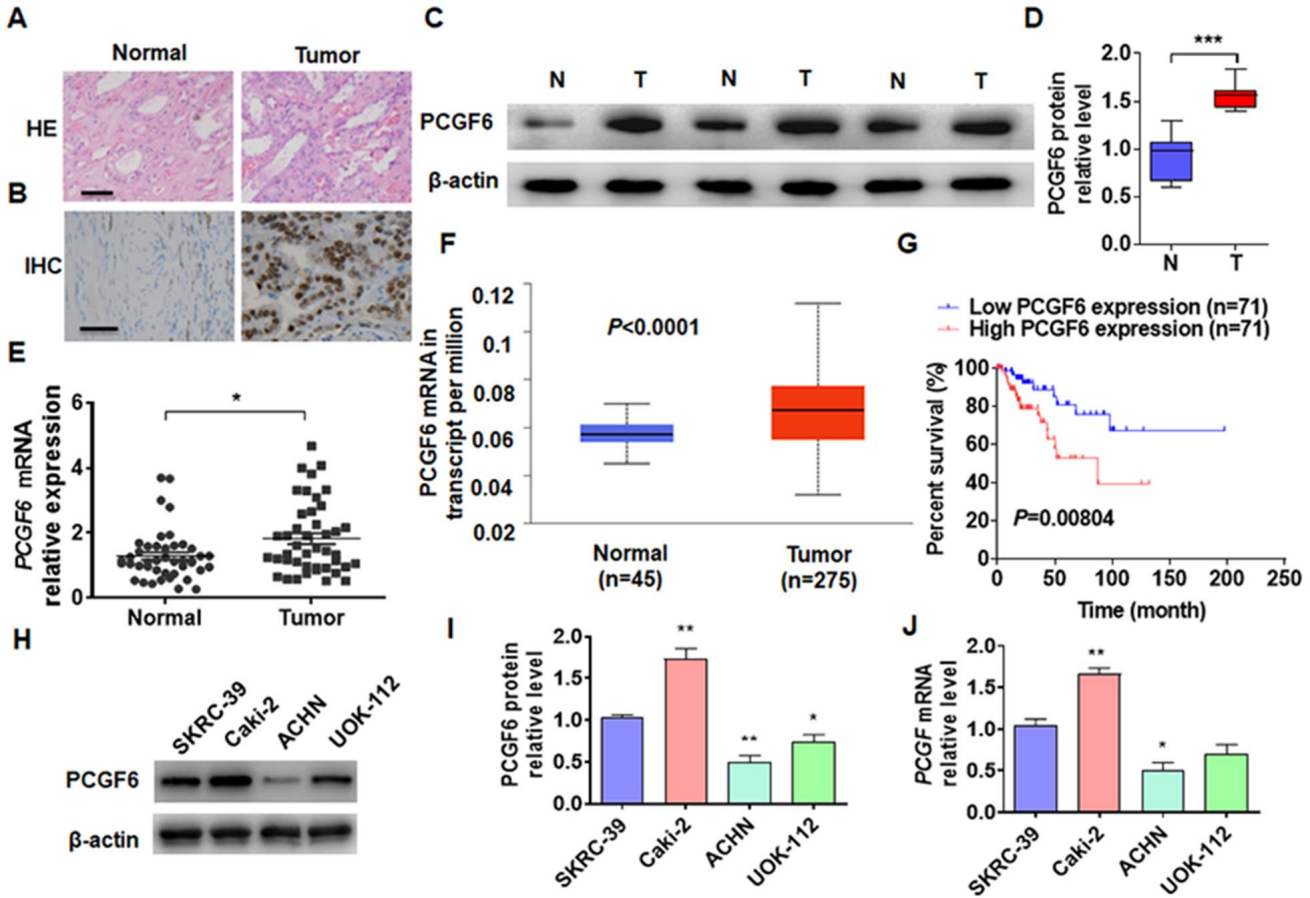
The upregulation of PCGF6 is strongly associated with poor prognosis of patients in pRCC. **A** H&E staining was used to confirm pRCC and healthy kidney tissues. Scale bar = 25 μm. **B** The expression of PCGF6 in cancer and healthy kidney tissues was examined by immunohistochemical (IHC) staining. Scale bar = 25 μm. **C** The protein expression of PCGF6 was detected in tumor (T) and normal (N) kidney tissues using Western blotting. **D** Quantitative Western blotting of **C**. **E** The mRNA level of PCGF6 was explored using RT-qPCR in tumor (*n* = 31) and healthy kidney (*n* = 31) tissues. **F** TCGA database was used to analyze mRNA expression of PCGF6.** G** Kaplan–Meier analysis of the relationship between the expression level of PCGF6 and patient prognosis in the TCGA database. **H** The protein expression of PCGF6 was measured using Western blotting in pRCC cell lines (i.e., SKRC-39, Caki-2, ACHN, and UOK-112). **I** Quantitative analysis of the data from Western blotting of **H**. **J** The mRNA level of PCGF6 was measured using RT-qPCR in the aforementioned cell lines. All data are from three independent experiments and are expressed as mean ± standard error. *p < 0.05, **p < 0.01 versus the corresponding controls

Studies reported that PCGF6 was closely related to stem cell differentiation and various tumor progression [29–31]. However, the expression and specific function of PCGF6 in pRCC remain unclear. First, H&E staining was performed to confirm the collected pRCC and healthy kidney tissues (Fig. 1a). Subsequently, the expression of PCGF6 in pRCC and healthy kidney tissues were tested through the immunohistochemical staining and Western blotting. The protein level of PCGF6 was significantly increased in pRCC tissues as compared to normal kidney tissues (Fig. 1b–d). The mRNA expression of PCGF6 was measured in pRCC and healthy kidney tissues using RT-qPCR. The mRNA expression of PCGF6 in pRCC tissue was consistent with its protein expression, and both were obviously higher than those in healthy kidney tissue (Fig. 1e). The pRCC data were analyzed in The Cancer Genome Atlas (TCGA) database and found that the mRNA level of PCGF6 was obviously higher in pRCC tissue than in healthy kidney tissue (Fig. 1f). The Kaplan–Meier correlation method was used to analyze the TCGA database. The results showed that higher PCGF6 mRNA expression in patients with pRCC could lead to poorer overall survival (Fig. 1g). A statistically significant relationship was found between PCGF6 expression and TNM stage, but not with other clinicopathologic factors, such as gender, age, and pT status (Table 1). Next, the expression of PCGF6 was examined in the pRCC cell line, and the results showed that the level of PCGF6 in the ACHN cell line was obviously lower than that in other cell lines, whereas the expression of PCGF6 in Caki-2 was higher (Fig. 1h–j). Therefore, in subsequent experiments, we will overexpress PCGF6 in the ACHN cell line and knock down PCGF6 in the Caki-2 cell line. These results suggested that the upregulation of PCGF6 might play a role in promotion of pRCC progression.

**Table 1:** Clinicopathological characteristics

Characteristics	Number of patients (%)	PCGF6 expression		
		Low (%)	High (%)	*p* value
No. of patients	31	16	15	
Age				
≤ 62	17	8 (47.06)	9 (52.94)	1.000
> 62	14	7 (50.00)	7 (50.00)	
Gender				
Male	21	10 (47.62)	11 (52.38)	0.704
Female	10	6 (60.00)	4 (40.00)	
Tumor size (cm)				
≤ 3	19	13 (68.42)	6 (31.58)	0.705
> 3	12	7 (55.56)	5 (44.44)	
pT status				
pT_1_–pT_2_	17	11 (64.71)	6 (35.29)	0.290
pT_3_–pT_4_	14	6 (42.86)	8 (57.14)	
pN status				
pN0	20	12 (60.00)	8 (40.00)	0.478
pN1–pN3	11	5 (45.45)	6 (54.55)	
TNM stage				
I–II	22	6 (27.27)	16 (72.73)	0.017
III–IV	9	7 (77.78)	2 (22.22)	

### PCGF6 facilitates cell proliferation in pRCC

**Fig. 2:**
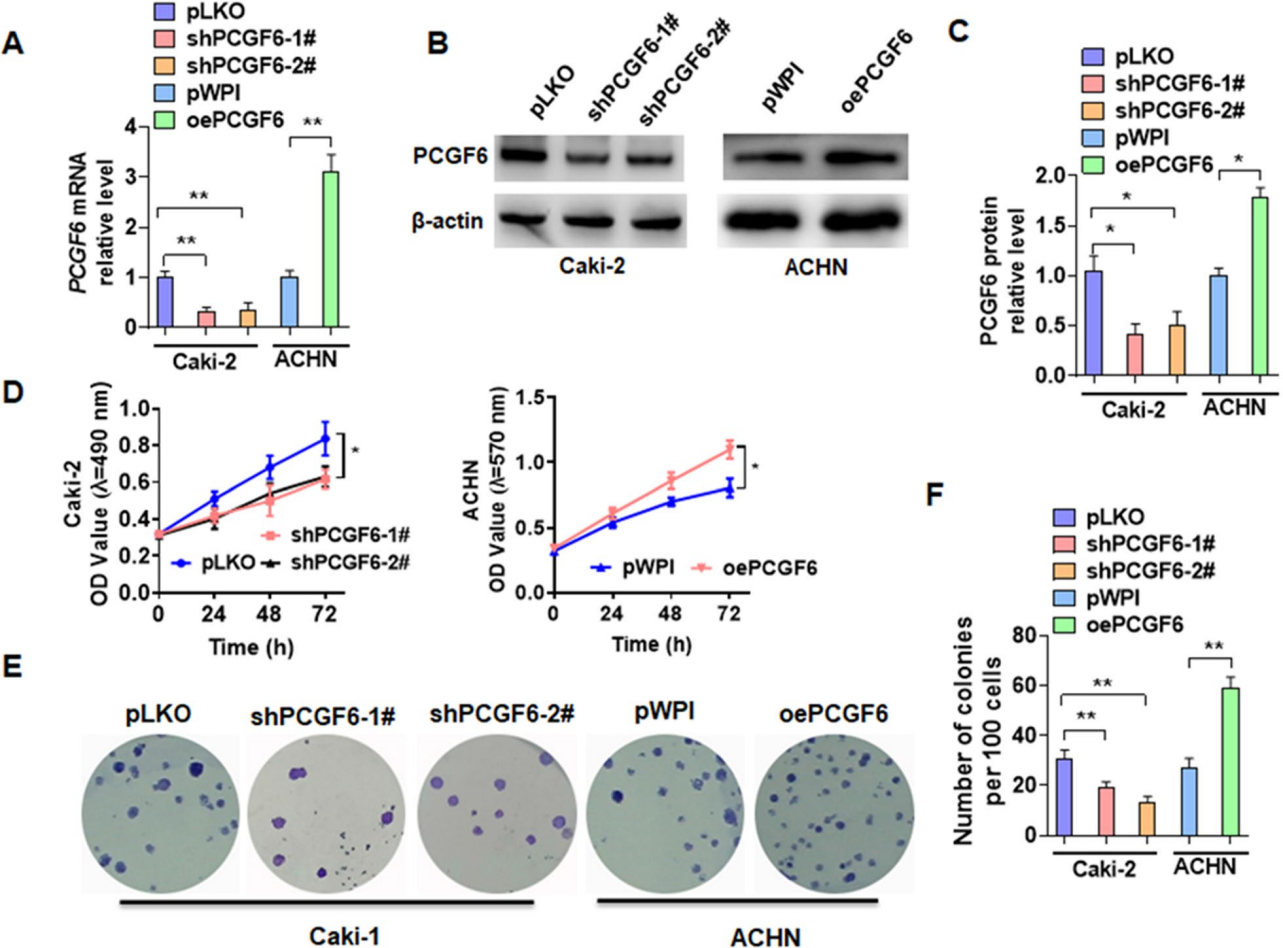
PCGF6 facilitates pRCC cell growth in vitro. **A** Caki-2 cells were transfected with two vectors of PCGF6 shRNAs (shPCGF6-1# and shPCGF6-2#), ACHN cells were transfected with PCGF6 overexpression vectors. RT-qPCR was conducted to measure the mRNA level of PCGF6. **B** The indicated vectors were transfected into cells as **A**, and the expression of PCGF6 was measured using Western blotting. **C** Quantitative analysis of Western blotting from **B**. **D**–**F** Cell transfection as **A**, and cell viability was detected using MTT (**D**) and colony formation assays (**E**, **F**). All data are from three independent experiments and are expressed as mean ± standard error. *p < 0.05, **p < 0.01 versus the corresponding controls

To investigate how PCGF6 plays function in pRCC, the loss-or-gain of function experiments were implemented in vitro. We first constructed a PCGF6 overexpression vector and two shRNAs that knocked down PCGF6. The transfection of oePCGF6 in ACHN cells markedly elevated the expression of PCGF6. However, shPCGF6 transfection significantly inhibited the expression of PCGF6 in Caki-2 cells (Fig. 2a–c). MTT assay was conducted to examine cell viability. The results showed that the depletion of PCGF6 inhibited the growth of Caki-2 cells, whereas the overexpression of PCGF6 in ACHN cells promoted cell proliferation (Fig. 2d). Colony formation assay showed similar results (Fig. 2e, f). These results showed that PCGF6 played a role in promoting the growth of pRCC cells.

### MAZ expression was upregulated in pRCC and was positively correlated with PCGF6 expression

**Fig. 3:**
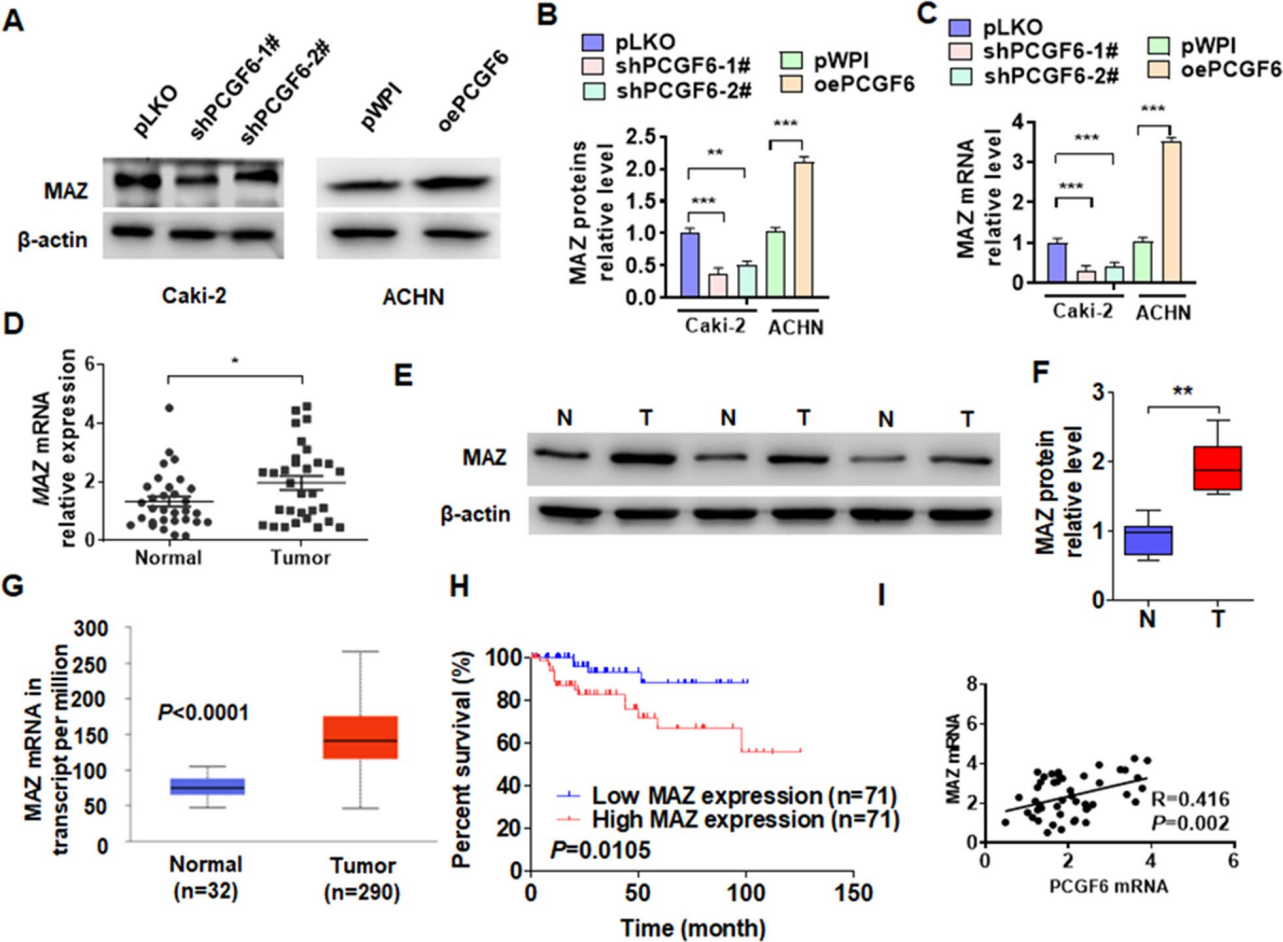
MAZ is downstream molecular of PCGF6 in pRCC and is positively correlated with PCGF6 expression. **A** Caki-2 cells were transfected with two vectors of PCGF6 shRNAs (shPCGF6-1# and shPCGF6-2#), ACHN cells were transfected with PCGF6 overexpression vectors. Western blot analysis was used to examine MAZ protein expression. **B** Quantitative analysis of Western blotting from **A**. **C** The indicated vectors were transfected into cells as **A**, and the expression of MAZ was measured using RT-qPCR. **D** The mRNA level of MAZ was measured using RT-qPCR in tumor (*n* = 31) and healthy kidney (*n* = 31) tissues. **E** The protein expression of MAZ was measured using Western blotting in pRCC tissues. **F** Quantitative analysis of the data from Western blotting of **E**. **G** TCGA database was used to analyze mRNA expression of MAZ. **H** Kaplan–Meier analysis of the relationship between the expression level of MAZ and patient prognosis in the TCGA database. **I** Correlation analysis was performed between MAZ and PCGF6 mRNA expression in pRCC tissues. All data are from three independent experiments and are expressed as mean ± standard error. *p < 0.05, **p < 0.01, ***p < 0.001 versus the corresponding controls

Our previous study found that MAZ was increased in ccRCC and the upregulation of MAZ facilitated tumor progression [24]. To explore whether PCGF6 promotes pRCC progression by regulating MAZ expression. We overexpressed and knocked down of PCGF6 and examined the MAZ expression. The results showed that depletion of PCGF6 significantly reduced MAZ protein and mRNA level, while overexpression of PCGF6 elevated MAZ expression (Fig. 3a–c). Next, we explored MAZ expression in pRCC tissues. As shown in Fig. 3d–f, both mRNA and protein expression of MAZ in pRCC tissues were obviously higher than those in healthy kidney tissue. The same results were obtained from pRCC data analyzed in the Cancer Genome Atlas (TCGA) database (Fig. 3g). The Kaplan–Meier correlation method was used to analyze MAZ expression in the TCGA database. The results revealed that higher MAZ mRNA expression in patients with pRCC could lead to poorer overall survival (Fig. 3h). In addition, both our PCR results and the data from TCGA indicated that MAZ expression was positively correlated with PCGF6 expression in pRCC tissues (Fig. 3i and Additional file 2: Fig. S1). Together, these results suggested that the upregulation of MAZ in pRCC might play a role in PCGF6-regulated pRCC progression.

### MAZ mediated PCGF6-regulated pRCC cells proliferation

**Fig. 4:**
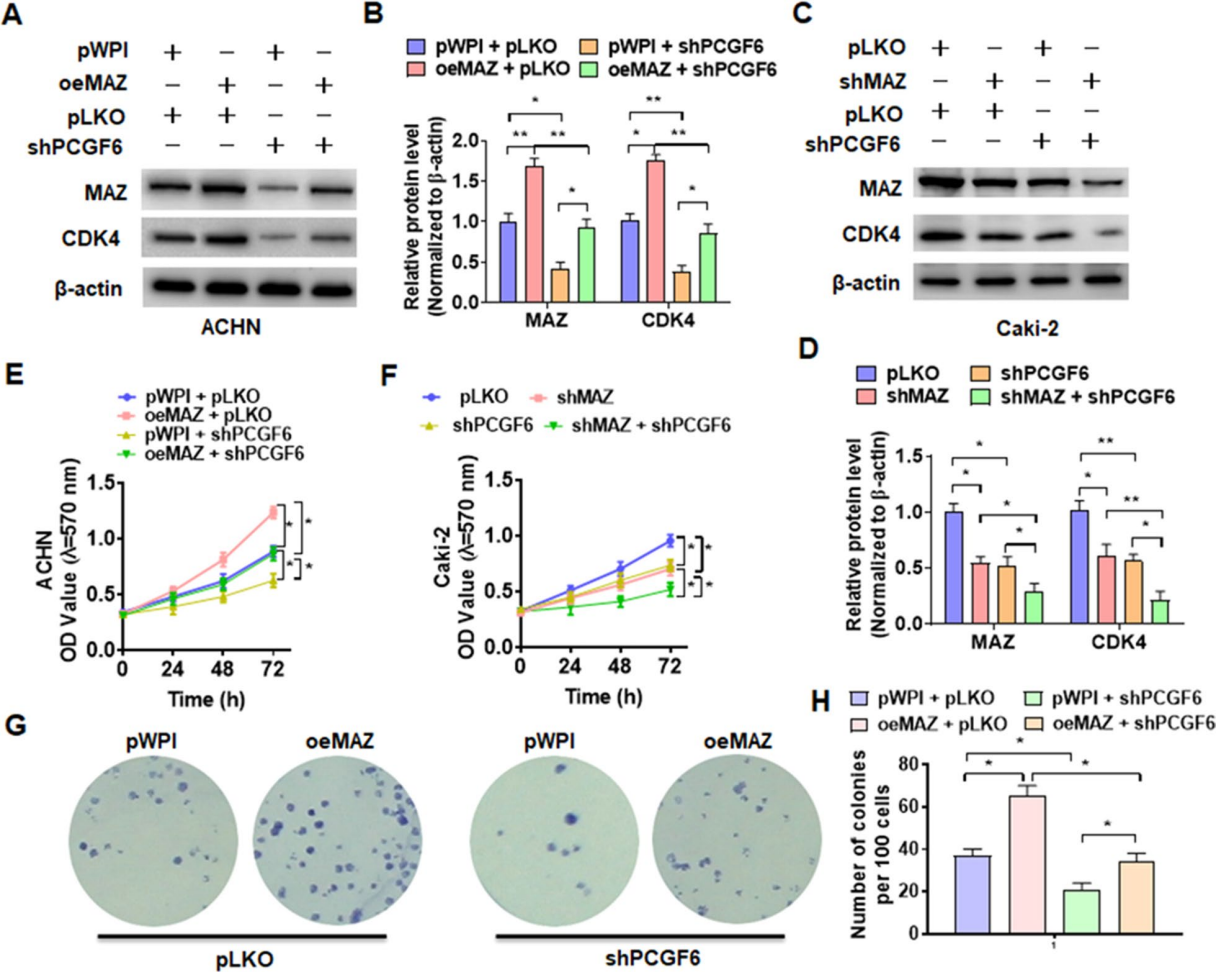
MAZ is involved in PCGF6-regulated pRCC cells proliferation. **A** ACHN cells were transfected with MAZ overexpression vector (oeMAZ) or PCGF6 shRNA or corresponding control vectors alone or together, and then Western blotting was used to examine the protein levels of MAZ and CDK4. **B** Quantitative analysis of Western blotting from **A**. **C** Caki-2 cells were transfected with shMAZ or shPCGF6 or pLKO control vector alone or together, then MAZ and CDK4 protein level were detected by Western blot. **D** Quantitative analysis of Western blotting from **C**. **E** ACHN cells were transfected as in **A**, and cell viability was detected using MTT. **F** Caki-2 cells were transfected as in **C**, and MTT was used to measure cell viability. **G**, **H** ACHN cells were transfected as in **A**, and colony formation assays was used to explore cell proliferation. All data are from three independent experiments and are expressed as mean ± standard error. *p < 0.05, **p < 0.01 versus the corresponding controls

To investigate whether MAZ is involved in PCGF6-regulated pRCC progression, we first transfected Caki-2 cells with MAZ shRNA and ACHN cells with its overexpression vector. Transfection with oeMAZ vector in ACHN cells markedly elevated the expression of MAZ. However, shMAZ transfection significantly inhibited the expression of MAZ in Caki-2 cells (Additional file 2: Fig. S2A–C). Next, ACHN cells were co-transfected with oeMAZ and shPCGF6 or their corresponding control vectors, and Western blot was used to detected MAZ and CDK4 proteins level. As shown in Fig. 4a, b, overexpression of MAZ significantly increased CDK4 protein expression. However, this promotion effect was partially offset in the depletion of PCGF6, simultaneously. Conversely, the depletion of MAZ reduced the expression of CDK4 in Caki-2 cells. However, this inhibition effect was enhanced in the depletion of both MAZ and PCGF6 (Fig. 4c, d). Subsequently, rescue experiments were used to detect the function of PCGF6/MAZ in pRCC cells growth. Overexpression of MAZ facilitated the ACHN cells proliferation, and this could be partially offset with suppression of PCGF6, simultaneously (Fig. 4e). In contrast, depletion of MAZ significantly depressed the growth of Caki-2 cells while simultaneous knockdown of MAZ and PCGF6 enhanced the inhibitory effect (Fig. 4f). The colony formation assays also obtained the same results (Fig. 4g, h). These results demonstrated that MAZ was involved in PCGF6-regulated pRCC cells proliferation.

### PCGF6 interacts with MAX/KDM5D and promotes MAZ promoter hypomethylation

**Fig. 5:**
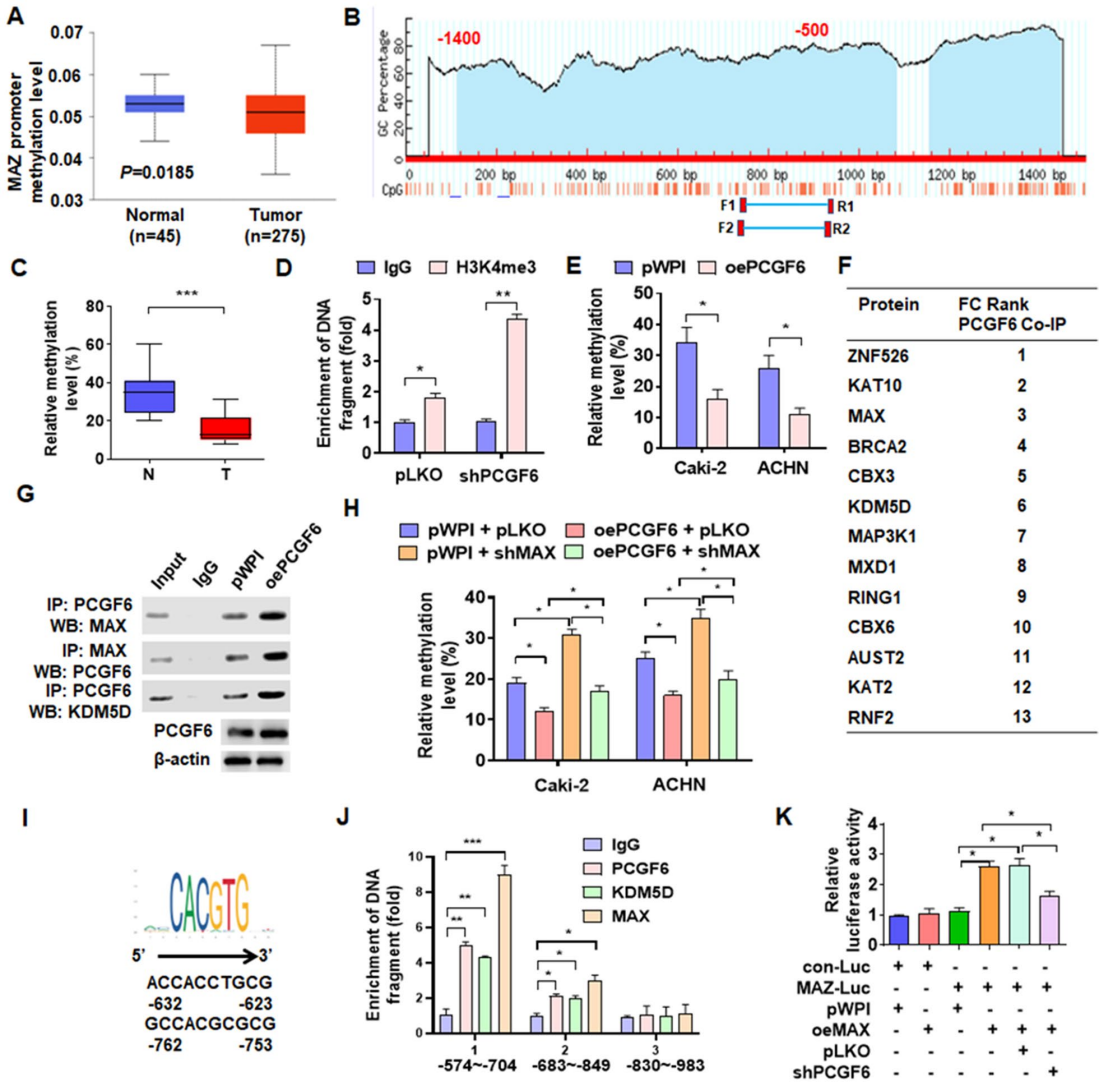
MAX recruits PCGF6 and KDM5D to the promoter region and facilitates MAZ hypomethylation. **A** TCGA data were used to determine MAZ promoter methylation levels in tumor and healthy kidney tissues. **B** Methprimer was used to analyze the CpG island within MAZ promoter (1400 bp). **C** DNA methylation of MAZ promoter was examined using bisulfite sequencing PCR (BSP). **D** Caki-2 cells were transfected with shPCGF6-1# (shPCGF6) or control vector, and then ChIP-PCR was used to explore the CpG isolate of MAZ promoter with IgG or H3K4me3 antibody.** E** DNA methylation in CpG island of the MAZ promoter was measured by bisulfite sequencing PCR (BSP) in Caki-2 and ACHN with indicated transfection. **F** Cells overexpressed with PCGF6 were immunoprecipitated with PCGF6 antibody and then analyzed using CoIP-MS. There are 13 proteins listed in the table that interact more strongly with PCGF6 after overexpression of PCGF6. **G** PCGF6, MAX, and KDM5D interaction were detected by the CoIP-Western blot. **H** After transfection of Caki-2 and ACHN with oePCGF6 or shMAX or corresponding control vectors, the DNA methylation condition of the CpG island of the MAZ promoter was examined using BSP. **I** Potential binding sites of MAX within CpG island of MAZ promoter were analyzed using the Ensembl and PROMO 3.0 websites. **J** With antibodies against PCGF6, KDM5D, and MAX, ChIP-PCR was used to determine the binding site of MAX/PCGF6/KDM5D complex on CpG island. **K** ACHN cells co-transfected with the indicated vectors were used for the luciferase reporter assays. *p < 0.05, **p < 0.01, ***p < 0.001 versus the corresponding controls

Demethylation of gene promoters play a critical role in RCC progression [32–34]. To investigate whether PCGF6 regulates MAZ expression by facilitating promoter demethylation, we first analyzed the modification of MAZ promoter methylation in the TCGA database. The analysis indicated that the methylation level of MAZ promoter in pRCC tissues was significantly lower than that in healthy kidney tissues (Fig. 5a). Next, we analyzed the CpG island of MAZ promoter (Fig. 5b), then MSP and bisulfite sequencing PCR (BSP) were performed to detect methylation level in CpG island. The BSP results indicated that the methylation level of MAZ promoter CpG was lower in pRCC tissues compare in normal kidney tissue (Fig. 5c). Previous study showed that PCGF6 associated with the H3K4me3 and negatively regulated its levels. To explore whether PCGF6 promoted the H3K4 histone demethylation, shPCGF6 was transfected into cells and examined the H3K4me3 methylation level in MAZ promoter by ChIP. The results showed that suppression of PCGF6 markedly increased the H3K4me3 methylation level (Fig. 5d). Additionally, overexpression of PCGF6 in both cell lines significantly suppressed the methylation levels of the MAZ promoter CpG island (Fig. 5e). A CoIP-mass spectrometry (CoIP-MS) was conducted to identify how PCGF6 promoted the demethylation of MAZ promoter. It was found that 13 proteins enhanced their interaction with PCGF6 in PCGF6-overexpressed cells (Fig. 5f, Additional file 3). Next, CoIP-combined Western blot verified that MAX and KDM5D interacted with PCGF6 (Fig. 5g). To identify whether MAX was involved in PCGF6-regulated demethylation of MAZ promoter, cells were co-transfected with oePCGF6 and shMAX vectors. The results revealed that depletion of MAX promoted the methylation of the MAZ promoter, which was partially offset by overexpressing PCGF6 (Fig. 5h). In addition, Correlation analysis shows that hyper-methylation of MAZ expression was inversely correlated with PCGF6 expression in pRCC tissues (Additional file 2: Fig. S3). Since MAX is a transcription factor, and we want to know whether MAX recruits PCGF6 and KDM5D to CpG island. Next, the MAX binding motif within the CpG island of MAZ promoter was analyzed and found two potential MAX binding motifs in CpG island (Fig. 5i). Subsequently, a ChIP-PCR assay was performed and showed that PCGF6, KDM5D, and MAX combine to the CpG island mainly located − 574 to − 704 bp among the MAZ promoter (Fig. 5j). The luciferase assay was performed in Caki-2 cells to investigate whether PCGF6, KDM5D, and MAX complex regulate the promoter activity of MAZ. We found that elevation of MAX expression clearly promoted the luciferase activity of the MAZ promoter. However, depletion of PCGF6 together could be reversed the promotion effect of elevated-MAX (Fig. 5k). In addition, the result showed that lacking MAX binding motifs clearly inhibited the luciferase activity of the MAZ promoter (Additional file 2: Fig. S4). Together, these findings suggested that one function of PCGF6 was to interact with MAX and KDM5D and exaltation of MAZ expression through histone demethylation.

### CDK4 is involves in MAZ-regulated pRCC progression

**Fig. 6:**
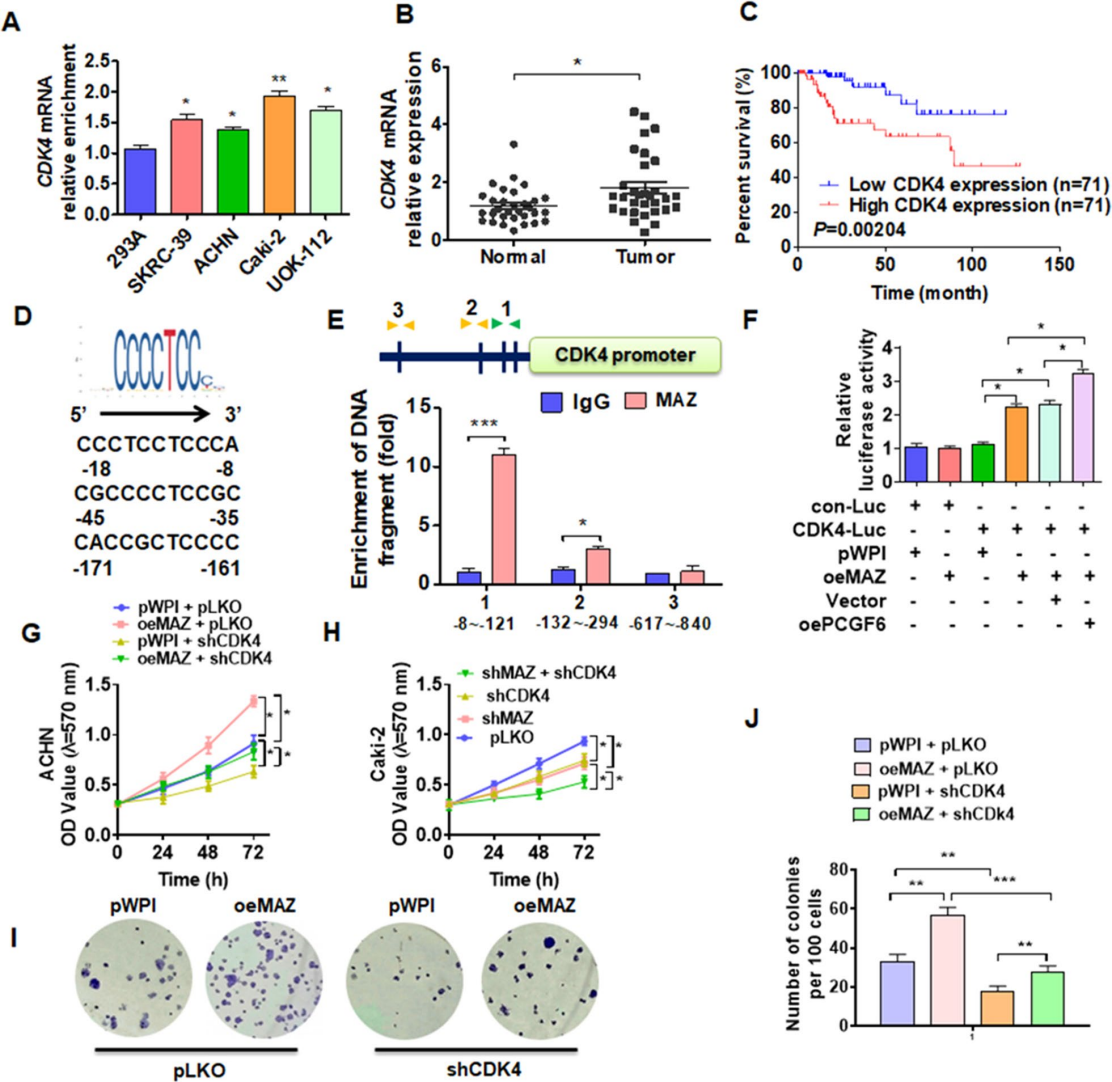
CDK4 is involved in MAZ-regulated pRCC cell proliferation. **A** RT-qPCR was used to examine CDK4 mRNA expression in different cell lines. **B** RT-qPCR was used to measure CDK4 mRNA levels in clinical tissues.** C** The Kaplan–Meier analysis was used to determine survival rates for patients with pRCC based on CDK4 levels. **D** The CDK4 promoter contains the potential binding site for MAZ. **E** ChIP-qPCR detected MAZ binding to CDK4 promoter region in Caki-2 cells. **F** Luciferase reporter assays were performed to detect CDK4 promoter activity in Caki-2 cells after cotransfected with indicated vectors. **G** ACHN cells were transfected with oeMAZ or shCDk4 or corresponding control vectors alone or together, and then cell viability was detected using MTT. **H** Caki-2 cells were transfected with shMAZ or shCDk4 or pLKO control vectors alone or together, and MTT was used to measure cell viability. **I**, **J** ACHN cells were transfected as in **G**, and colony formation assays was used to explore cell proliferation. All data are from three independent experiments and are expressed as mean ± standard error. *p < 0.05, **p < 0.01, ***p < 0.001 versus the corresponding controls

CDK4 as an important marker gene for cell proliferation may be involved in MAZ-regulated cell proliferation, and then we measured CDK4 expression in different cell lines. As shown in Fig. 6a, the mRNA level of CDK4 was upregulated in pRCC cell lines compared with that in 293A cells. Next, we examined CDK4 expression in pRCC tissues and found that the expression of CDK4 was significantly increased (Fig. 6b). The higher CDK4 mRNA expression in patients with pRCC predicted poor overall survival (Fig. 6c). Subsequently, the binding sites of MAZ in CDK4 promoter were analyzed to confirm whether MAZ transcriptionally regulates CDK4 expression, and three potential bound motifs were found in this region (Fig. 6d). In addition, ChIP-PCR showed that MAZ was predominantly bound to the proximal site (− 8 to − 121 nt) of the transcription within CDK4 promoter (Fig. 6e). Luciferase assay demonstrated that MAZ increased the luciferase activity of CDK4 promoter, and overexpression of PCGF6 simultaneously enhanced this effect (Fig. 6f). And the result showed that lacking MAZ binding motifs clearly inhibited the luciferase activity of the CDK4 promoter (Additional file 2: Fig. S5). Rescue experiments were used to detect the function of MAZ/CDK4 in pRCC cells growth. Overexpression of MAZ facilitated the ACHN cells growth, and this effect could be offset with suppression of CDK4, simultaneously (Fig. 6g). In contrast, deletion of MAZ significantly depressed the growth of Caki-2 cells while simultaneous knockdown of MAZ and CDK4 enhanced the inhibitory effect (Fig. 6h). In parallel, we obtained the similarity results from the colony formation assays (Fig. 6i, j). Together, these results revealed that MAZ/CDK4 axis regulated pRCC cells proliferation.

### Disruption of PCGF6/MAZ/CDK4 axis inhibits pRCC xenograft progress in vivo

**Fig. 7:**
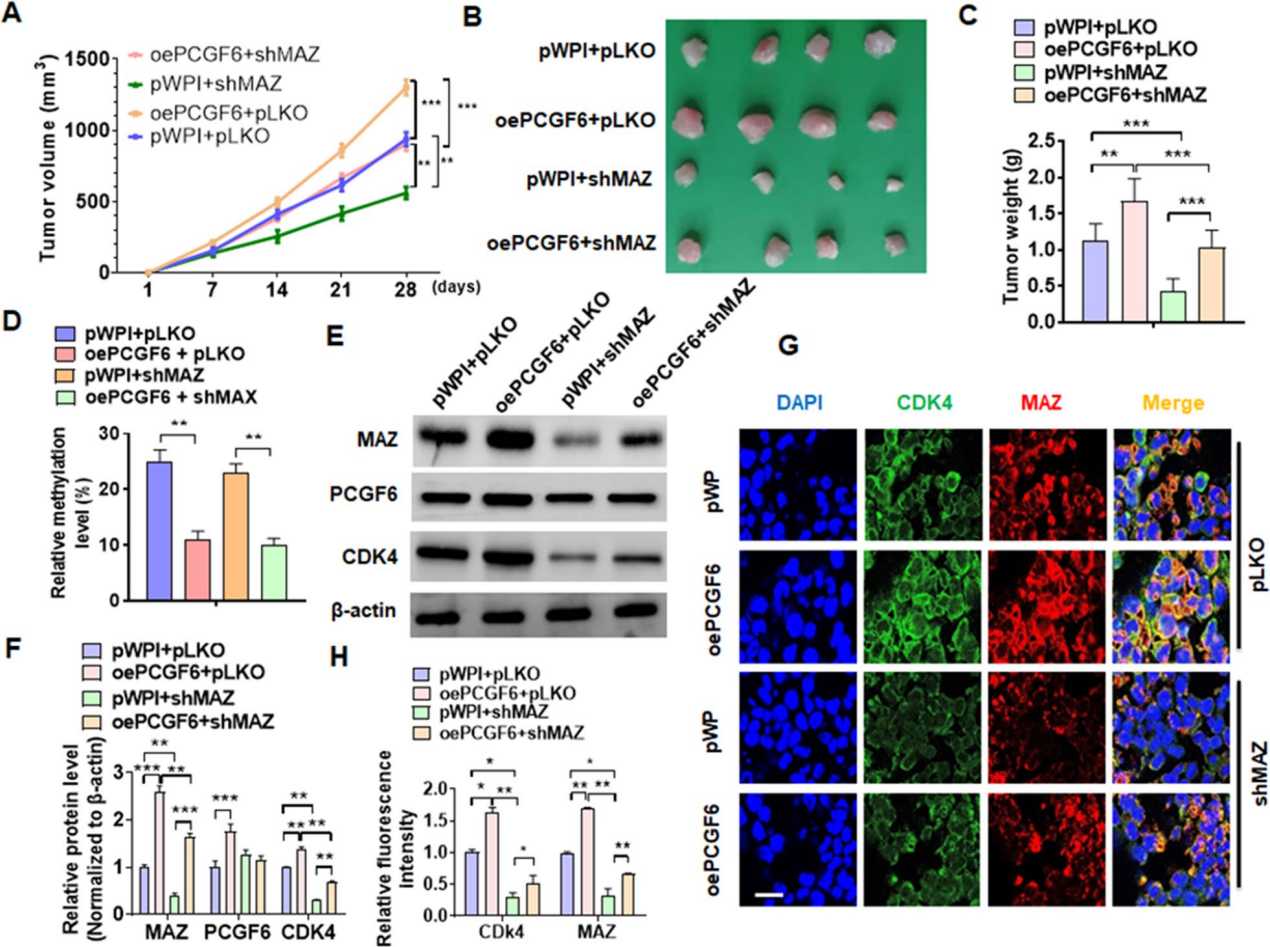
The disruption of PCGF6/MAZ/CDK4 axis inhibits pRCC xenograft progression in vivo. **A** Tumor volumes were measured in nude mice with xenograft of Caki-2 cells with stable deletion of MAZ or overexpression of PCGF6, alone or together. **B** The representative tumor size of all mice is presented. **C** Measuring wet weight of xenograft tumors. **D** The DNA methylation condition of the CpG island of the MAZ promoter in xenograft tumors were examined using BSP. **E** The protein levels of MAZ, PCGF6, and CDK4 were measured in tumors using Western blotting. **F** Quantitative analysis of Western blotting data from **E**. **G** Double immunofluorescence staining measures the level of MAZ and CDK4 in xenograft tumors. **H** Quantitative analysis of fluorescence intensity of **G**. All data are from three independent experiments and are expressed as mean ± standard error. *p < 0.05, **p < 0.01, ***p < 0.001 versus the corresponding controls

**Fig. 8:**
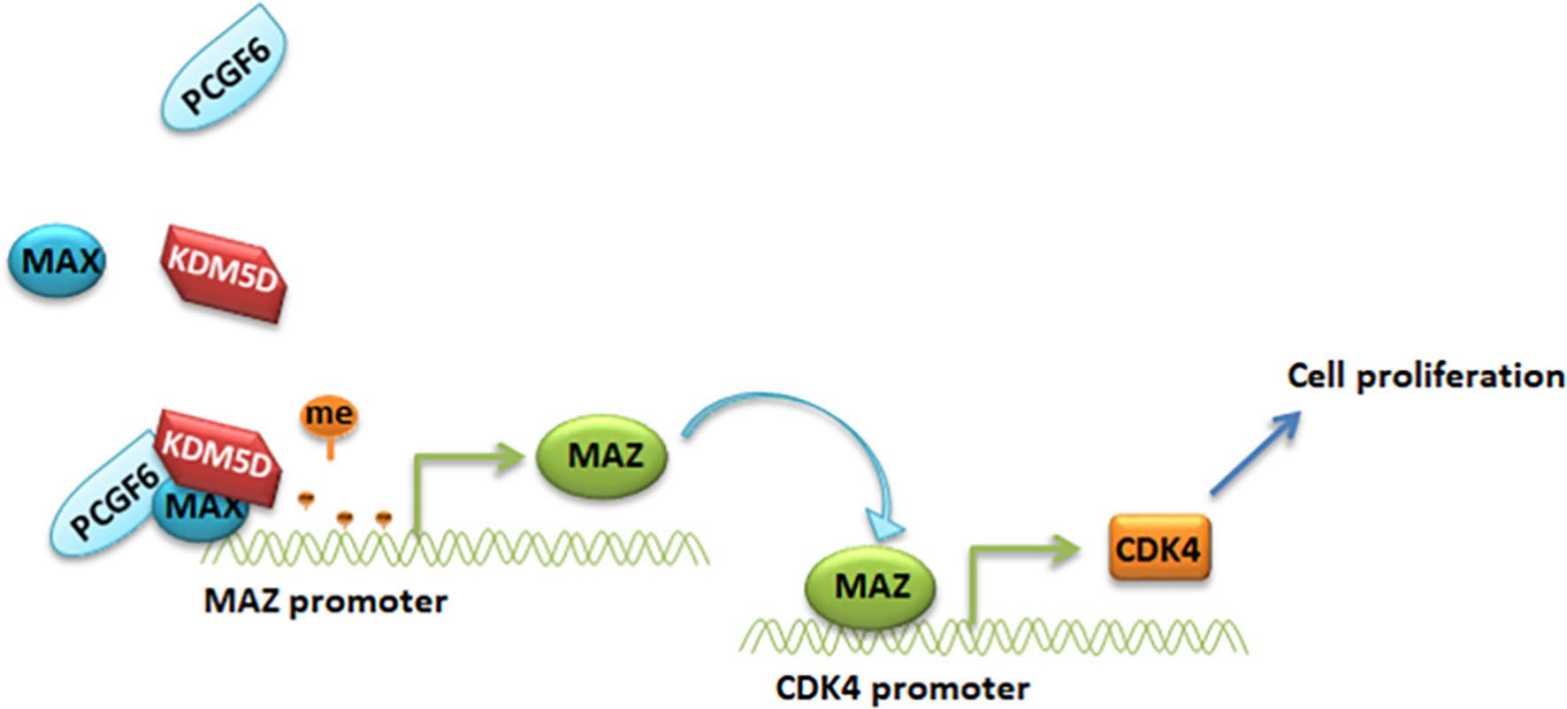
Proposed model for PCGF6/MAZ/CDK4 regulation of pRCC progression

A xenograft model was developed and employed to examine whether PCGF6/MAZ/CDK4 axis regulate pRCC cell growth in vivo. MAZ depletion significantly depressed tumors growth in nude mice while overexpression of PCGF6 simultaneous partial offset the effect of suppression (Fig. 7a, b). The average wet tumor weight after resection confirmed these findings (Fig. 7c). To confirm whether PCGF6 is also involved in regulating hypomethylation of MAZ promoter in vivo, we tested the level of methylation in xenograft tumor tissue. The results showed that overexpression of PCGF6 markedly decreased level of methylation of MAZ promoter. However, depletion of MAZ did not affect PCGF6-regulated methylation of MAZ promoter (Fig. 7d). Next, Western blot also demonstrated that xenograft tumors derived from MAZ-depleted cells exhibited marked downregulation of MAZ and CDK4 expression, and this reduction was partially reversed by simultaneous overexpression of PCGF6 (Fig. 7e, f). The results of immunofluorescence staining also confirmed this finding (Fig. 7g, h). Taken together, these results suggested that blocking the PCGF6/MAZ/CDK4 axis inhibited in vivo pRCC progression (Fig. 8).

## Discussion

The role of PCGF6/MAZ/CDK4 axis in regulating pRCC tumorigenesis was investigated. It was found from the clinical samples and TCGA database that the expression of MAZ in pRCC tissues was obviously elevated, and the upregulation of MAZ in patients was predicted with a poor prognosis. MAZ was reported to act as an oncogene by promoting the growth of pRCC cells. MAZ promoter was found to be hypomethylated, and PCGF6 regulated the demethylation of H3K4me3 in CpG island of MAZ promoter. Mechanistically, MAX recruited PCGF6 and KDM5D to CpG island of the MAZ promoter and formed a complex in which PCGF6 interacted with MAX and KDM5D to induce H3K4me3 demethylation at the MAZ promoter, thus leading to DNA hypomethylation. This study results showed that PCGF6/MAZ/CDK4 axis promoted pRCC growth.

Cyclin-dependent kinases (CDKs) are essential chaperone kinases for cells to complete the cell cycle process [35]. Overactive CDKs in tumors are believed to be one of the hallmarks of important malignancies that drive cell division and generate sustained proliferative signals [36]. The cell cycle is regulated by a variety of proteins, among which D-type cyclin and its related CDKs (CDK4) play a crucial role in the cell cycle mechanism. Studies have shown that phosphorylation and inactivation of retinoblastoma protein (RB) can drive the transition from G1 to S phase [37]. The cyclin D–CDK4 complex is a major integrator of various mitogenic and antimitotic signals. It phosphorylates SMAD3 in a cell cycle-dependent manner and inhibits its transcriptional activity [38]. Many human cancers have genomic or transcriptional abnormalities that activate CDK4/6, and inhibition of CDK4/6 has become a potential target for the development of antitumor drugs [39]. Therefore, studying mechanism of action of CDK4/6 in tumors may provide a theoretical basis for the development of more targeted therapies. Zhang et al. reported that HMGB1 is an important factor in the mechanism of tamoxifen resistance and serves as a predictor of the therapeutic effect of CDK4/6 inhibitors in breast cancer. [40]. Ribociclib suppression of CDK4/6–cyclin D-Rb signal path enhances chemotherapy and immunotherapy in RCC [41]. This study showed that MAZ-promoted pRCC progression through transcriptional regulation of CDK4. Therefore, targeting the PCGF6/MAZ/CDK4 axis may be one of the effective ways to treat pRCC.

MAZ is involved in the progression and metastasis of multiple cancer types and the expression is upregulated in various cancers [15, 22, 42]. Elevated expression of MAZ reported to enhance the growth and metastasis of prostate cancer by increasing the expression of androgen receptors [43]. MAZ promotes bone metastasis of prostate cancer through transcriptional promotion of the KRas/RalGEFs signal path [15]. MAZ is a downstream molecule of Cyr61/CCN1, which expands the invasion of pancreatic cancer cells through CRAF-ERK signaling [22]. MAZ is reported to be upregulated in ccRCC tissues and cells, and this increases ccRCC cell growth and tumor progression (data not shown). However, the role and biological function of MAZ in the clinical treatment of pRCC remains unclear. The results of this study showed that the level of MAZ in pRCC tissue was higher than that in healthy kidney tissue, and the expression of MAZ was positively correlated with the overall survival rate of patients with pRCC. The expression of MAZ was found to be downregulated in ACHN, whereas it was upregulated in the Caki-2 and UOK-112 cell lines. It was speculated that as ACHN was a migrating cell carcinoma, it was different from cancer cells in situ. The difference in gene expression between carcinoma in situ and migrating cell carcinoma in pRCC needs to be explored.

## Supplementary Information

**Additional file 1: Table S1**. Primers used in the study.

**Additional file 2. Fig. S1**: **A** Correlation analysis was performed between MAZ and PCGF6 mRNA expression in pRCC tissues from the data of TCGA. **Fig. S2**: RT-qPCR and Western blot were used to verify MAZ expression. **A** Caki-2 cells were transfected with MAZ shRNAs and ACHN cells were transfected with MAZ overexpression vectors. RT-qPCR was conducted to measure the mRNA level of MAZ. **B** The indicated vectors were transfected into cells as **A**, and the expression of MAZ was measured using Western blotting. **C** Quantitative analysis of Western blotting from **B**. **Fig. S3**: Correlation analysis was performed between MAZ promoter hypo-methylation and PCGF6 mRNA expression in pRCC tissues. **Fig. S4**: ACHN cells co-transfected with the indicated vectors were used for the luciferase reporter assays. **p < 0.01 versus the corresponding controls. **Fig. S5**: Luciferase reporter assays were performed to detect CDK4 promoter activity in Caki-2 cells after cotransfected with indicated vectors.

**Additional file 3**. The proteins interacting with PCGF6 were analyzed by mass spectrometry.

## Data Availability

The data that support the findings of this study are available from the corresponding author upon reasonable request.
