# Supplementary material for: PCGF6/MAX/KDM5D facilitates MAZ/CDK4 axis expression and pRCC progression by hypomethylation of the DNA promoter
Source: Epigenetics Chromatin. 2023 Mar 9;16:9. doi: 10.1186/s13072-023-00483-w (PMC9996882; doi:10.1186/s13072-023-00483-w)
Supplement: Supplementary file 1 — Additional file 1: Table S1. Primers used in the study. [file 13072_2023_483_MOESM1_ESM.docx]

**Supplementary table 1** primers used in the study

| Name | Sequcence 5’ to 3’ |
| --- | --- |
| MAZ-F1 | GAAGAACCATGCCTGCGAGATGTG |
| MAZ-R1 | GCTGCCTCACATTTCTCACATTTGAAG |
| ASH1L-F | AGCGCCCATTCATGTTGTG |
| ASH1L-R | TTGGGGGAACACTCAGCAAA |
| MECOM-F | GGCTAGATTGCTTATTCATAGGGC |
| MECOM-R | CTGATCATAACAGCCAGCGA |
| NSD1-F | TGCAGCCAAGATGCAGTGTA |
| NSD1-R | AATCCAAGGCACTCCAGGTG |
| PRDM16-F | GCTGTCCCTTTCCGAAGACA |
| PRDM16-R | TCGGAATGAACCATGGTGGG |
| SETMAR-F | GCGGAGTTTAAGGAGAAGCC |
| SETMAR-R | AGTACAGAGAACTTCCTTTCGG |
| SETDB2-F | TGGACTTGTCTTTTGGTTGGAC |
| SETDB2-R | TGGTGGCAGACCCATCTTTG |
| SUV39H2-F | AAGCTCTACAAGATGGCGGC |
| SUV39H2-R | AAGCAGTAACGGGCACTTCA |
| RSBN1-F | GACATACTGCTGTGGCACCT |
| RSBN1-R | TGGTCCGAGGTAGGTACTGG |
| ATF7-F | TATGGGAGACGACAGACCGT |
| ATF7-R | TTGGGGTAACCTCCTCCACT |
| KDM2B-F | GACGCAAGCGGCTCAAAC |
| KDM2B-R | TCTCAGGCTCCGACTTGATG |
| KDM4A-F | TTCTCCGCCACAAGATGACC |
| KDM4A-R | AGCTCTTGGAGGCAGTTCAC |
| PCGF6-F | TGTTCCACAGCCAGTCCCTT |
| PCGF6-R | TGCACGTCGGATTTCCCTTA |
| GAPDH-F | ATGAATGGGCAGCCGTTAGG |
| GAPDH-R | TGGAATTTGCCATGGGTGGA |
| si-NSD1-F | CCUGGAACUUCAUCAUCAUUU |
| si-NSD1-R | AUGAUGAUGAAGUUCCAGGUU |
| si-SETDB2-F | GUGGCCUCGAGCAUAUAAUUU |
| si-SETDB2-R | AUUAUAUGCUCGAGGCCACUU |
| si-SUV39H2-F | GGUCCUGAUUGUCCCAAUAUU |
| si-SUV39H2-R | UAUUGGGACAAUCAGGACCUU |
| si-PCGF6-F | GCGCCUGAUUAAUCUCUCUUU |
| si-PCGF6-R | AGAGAGAUUAAUCAGGCGCUU |
|  |  |
|  |  |
