# Supplementary material for: PCGF6/MAX/KDM5D facilitates MAZ/CDK4 axis expression and pRCC progression by hypomethylation of the DNA promoter
Source: Epigenetics Chromatin. 2023 Mar 9;16:9. doi: 10.1186/s13072-023-00483-w (PMC9996882; doi:10.1186/s13072-023-00483-w)
Supplement: Supplementary file 2 — Additional file 2. Fig. S1: A Correlation analysis was performed between MAZ and PCGF6 mRNA expression in pRCC tissues from the data of TCGA. Fig. S2: RT-qPCR and Western blot were used to verify MAZ expression. A Caki-2 cells were transfected with MAZ shRNAs and ACHN cells were transfected with MAZ overexpression vectors. RT-qPCR was conducted to measure the mRNA level of MAZ. B The indicated vectors were transfected into cells as A, and the expression of MAZ was measured using Western blotting. C Quantitative analysis of Western blotting from B. Fig. S3: Correlation analysis was performed between MAZ promoter hypo-methylation and PCGF6 mRNA expression in pRCC tissues. Fig. S4: ACHN cells co-transfected with the indicated vectors were used for the luciferase reporter assays. **p < 0.01 versus the corresponding controls. Fig. S5: Luciferase reporter assays were performed to detect CDK4 promoter activity in Caki-2 cells after cotransfected with indicated vectors. [file 13072_2023_483_MOESM2_ESM.docx]

**PCGF6/MAX/KDM5D facilitates MAZ/CDK4 axis expression and pRCC progression by hypomethylation of the DNA promoter**

Meng Zhu^1#^, Ruo-Nan Zhang^2^, Hong Zhang^1^, Chang-bao Qu^1,^ Xiao-chong Zhang^4^, Li-Xin Ren^1^, Zhan Yang^1,3^, and Jun-Fei Gu^1^*

^1^Department of Urology, The Second Hospital of Hebei Medical University, 215 Heping W Rd, Shijiazhuang 050000, China; ^2^School of Chinese Integrative Medicine, Hebei Medical University, Shijiazhuang, Hebei, China. ^3^Molecular Biology Laboratory, Talent and Academic Exchange Center, The Second Hospital of Hebei Medical University, Shijiazhang, China; ^4^Clinical Laboratory, Xingtai People's Hospital, Xingtai, China.

Running title: PCGF6/MAX/KDM5D facilitates pRCC progression

*** Corresponding author**

Jun-Fei Gu, Department of Urology, The Second Hospital of Hebei Medical University, 215 Heping West Road, Shijiazhuang 050000, China.

Phone: 86311-6600-2850

Fax: 86311-6600-2850

E-mail: [gujunfeiey@163.com](mailto:gujunfeiey@163.com); Junfei_Gu2020@hebmu.edu.cn

**Supplementary material**


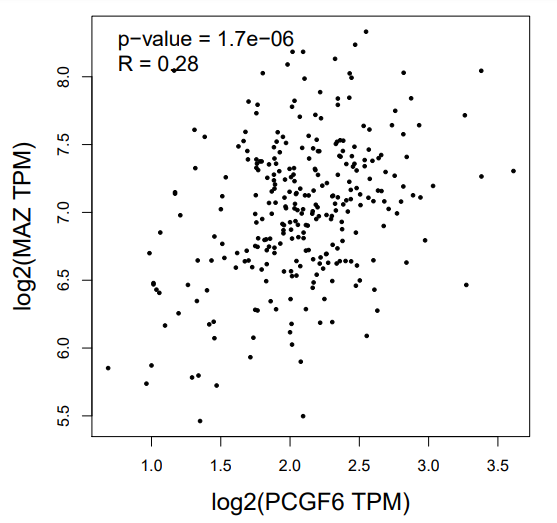


**Supplementary Figure 1. (A)**Correlation analysis was performed between MAZ and PCGF6 mRNA expression in pRCC tissues from the data of TCGA.

**C**

**A**

**B**

**ACHN**

**Caki-2**

**MAZ**

**β-actin**

**pLKO**

**shMAZ**

**pWPI**

**oeMAZ**


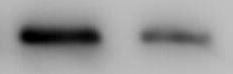

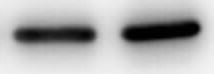

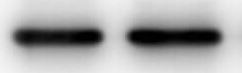

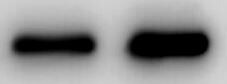


**Supplementary figure 2.** RT-qPCR and Western blot were used to verify MAZ expression. (A) Caki-2 cells were transfected with MAZ shRNAs and ACHN cells were transfected with MAZ overexpression vectors. RT-qPCR was conducted to measure the mRNA level of MAZ. (B) The indicated vectors were transfected into cells as (A), and the expression of MAZ was measured using Western blotting. (C) Quantitative analysis of Western blotting from (B).

**Supplementary Figure 3.** Correlation analysis was performed between MAZ promoter hypo-methylation and PCGF6 mRNA expression in pRCC tissues.

**Supplementary Figure 4.** ACHN cells co-transfected with the indicated vectors were used for the luciferase reporter assays. **p < 0.01 vs. the corresponding controls.

**Supplementary Figure 5.** Luciferase reporter assays were performed to detect CDK4 promoter activity in Caki-2 cells after cotransfected with indicated vectors.
